# Anaerobic fixed-target serial crystallography

**DOI:** 10.1107/S2052252520010374

**Published:** 2020-08-21

**Authors:** Patrick Rabe, John H. Beale, Agata Butryn, Pierre Aller, Anna Dirr, Pauline A. Lang, Danny N. Axford, Stephen B. Carr, Thomas M. Leissing, Michael A. McDonough, Bradley Davy, Ali Ebrahim, Julien Orlans, Selina L. S. Storm, Allen M. Orville, Christopher J. Schofield, Robin L. Owen

**Affiliations:** a Chemistry Research Laboratory, University of Oxford, 12 Mansfield Road, Oxford OX1 3TA, United Kingdom; b Diamond Light Source, Harwell Science and Innovation Campus, Didcot, Oxfordshire OX11 0DE, United Kingdom; c Research Complex at Harwell, Rutherford Appleton Laboratory, Didcot Oxfordshire OX11 0FA, United Kingdom; d School of Life Sciences, University of Essex, Wivenhoe Park, Colchester, Essex CO4 3SQ, United Kingdom; e UMR0203, Biologie Fonctionnelle, Insectes et Interactions, Institut National des Sciences Appliquées de Lyon, Institut National de Recherche pour l’Agriculture, l’Alimentation et l’Environnement, University of Lyon, Villeurbanne F-69621, France

**Keywords:** anaerobic crystallization, oxygen-employing enzymes, penicillin biosynthesis, 2-oxoglutarate/α-ketoglutarate oxygenases, serial crystallography

## Abstract

Cryogenic X-ray diffraction is a powerful tool for crystallographic studies on enzymes including oxygenases and oxidases. Amongst the benefits that cryo-conditions (usually employing a nitro­gen cryo-stream at 100 K) enable, is data collection of di­oxy­gen-sensitive samples. Although not strictly anaerobic, at low temperatures the vitreous ice conditions severely restrict O_2_ diffusion into and/or through the protein crystal. Cryo-conditions limit chemical reactivity, including reactions that require significant conformational changes. By contrast, data collection at room temperature imposes fewer restrictions on diffusion and reactivity; room-temperature serial methods are thus becoming common at synchrotrons and XFELs. However, maintaining an anaerobic environment for di­oxy­gen-dependent enzymes has not been explored for serial room-temperature data collection at synchrotron light sources. This work describes a methodology that employs an adaptation of the ‘sheet-on-sheet’ sample mount, which is suitable for the low-dose room-temperature data collection of anaerobic samples at synchrotron light sources. The method is characterized by easy sample preparation in an anaerobic glovebox, gentle handling of crystals, low sample consumption and preservation of a localized anaerobic environment over the timescale of the experiment (<5 min). The utility of the method is highlighted by studies with three X-ray-radiation-sensitive Fe(II)-containing model enzymes: the 2-oxoglutarate-dependent l-arginine hy­droxy­lase VioC and the DNA repair enzyme AlkB, as well as the oxidase isopenicillin N synthase (IPNS), which is involved in the biosynthesis of all penicillin and cephalosporin antibiotics.

## Abstract 2

An effective and simple-to-implement approach for anaerobic room-temperature data collection is described and demonstrated by application to dioxygen utilizing enzymes.

## 1. Introduction   

Enzyme-catalysed reactions using atmospheric di­oxy­gen are manifested in most kingdoms of life and have fundamental roles in many aspects of biology. Metal and/or organic cofactor-dependent enzymes catalyse the vast majority of O_2_ dependent reactions, many of which harness the chemical potential energy held within the O_2_ molecule to catalyse some of the most challenging reactions in biology (Huijbers *et al.*, 2014 ▸; Jasniewski & Que, 2018 ▸; Meier *et al.*, 2018 ▸; Huang & Groves, 2018 ▸; Goudarzi *et al.*, 2020 ▸). The importance of oxygenase/oxidase-related research in human biology is exemplified in work identifying the roles of 2-oxoglutarate (2OG) dependent protein hy­droxy­lases as sensors in the response to hypoxia in animals which ultimately led to the 2019 Nobel Prize for Medicine (http://www.nobelprize.org/prizes/medicine/). Identified roles of 2OG dependent oxygen­ases include collagen biosynthesis, regulation of gene expression, lipid metabolism, DNA repair, ribosome modification and the biosynthesis of secondary metabolites (Wilkins *et al.*, 2015 ▸; Schofield & Ratcliffe, 2005 ▸; Schofield & Hausinger, 2015 ▸). In plants and bacteria, members of the 2OG oxygenase structural family catalyse the formation of signalling molecules, notably ethyl­ene (Zhang *et al.*, 2002 ▸; Martinez *et al.*, 2017 ▸). Other key roles of 2OG dependent oxygenases are in nucleic acid/chromatin modifications (Falnes *et al.*, 2002 ▸; Trewick *et al.*, 2002 ▸; Yang *et al.*, 2008 ▸; Yi *et al.*, 2010 ▸). AlkB catalyses the repair of methyl­ation-induced DNA and RNA damage by methyl-group oxidation (Trewick *et al.*, 2002 ▸; Yang *et al.*, 2008 ▸; Yi *et al.*, 2010 ▸), resulting in a hemiaminal intermediate (Yang *et al.*, 2008 ▸) that fragments to give formaldehyde and the repaired DNA/RNA base. Other 2OG dependent de­methyl­ases, *e.g.* members of the JmjC KDM family, play important roles in the regulation of the genome by controlling the methyl­ation levels of histones and DNA. Some of these enzymes are therefore drug targets for the treatment of cancer and other diseases (Helin & Dhanak, 2013 ▸).

Since the solution of the first structure of an enzyme from the 2OG oxygenase structural family under anaerobic cryogenic conditions, isopenicillin N synthase (IPNS) (Roach *et al.*, 1997 ▸), crystallographic methods have improved and anaerobic cryo-crystallography now plays an important role within the oxygenase/oxidase structural biology field as a whole (Senda & Senda, 2018 ▸). The anaerobic chambers required to grow oxygenase crystals in their pre-di­oxy­gen binding state are widely available (Roach *et al.*, 1996 ▸). Cryo-cooling an anaerobically grown protein crystal to 100 K (often) appears to (substantially) maintain an anaerobic state even when crystals are exposed to atmospheric di­oxy­gen during data collection [see the work by Cedervall *et al.* (2010 ▸) for a notable exception]. However, a room temperature (RT), low-dose method to analyse oxygenase crystals would be particularly useful given that most oxygenases employ radiation-sensitive metal cofactors [*e.g.* Fe(II)] and their catalytic cycles involve conformational changes, raising the possibility of differences in binding modes between 100 K and more physiologically relevant temperatures (Keedy *et al.*, 2018 ▸).

RT crystallographic techniques that maintain an anaerobic environment for di­oxy­gen-dependent enzymes are challenging and largely unexplored. Some of the early methods were rather cumbersome and required sealing a capillary-mounted sample with either wax or ep­oxy resin (Roach *et al.*, 1996 ▸; Orville *et al.*, 1997 ▸). The advent of X-ray free-electron laser (XFEL) light sources has spurred the development of general RT methods at both XFELs and synchrotrons (Roedig *et al.*, 2015 ▸; Botha *et al.*, 2015 ▸; Weinert *et al.*, 2017 ▸; Ebrahim, Moreno-Chicano *et al.*, 2019 ▸). XFEL endstations lend themselves to anaerobic environments as the sample interaction point is often under either vacuum or 95% helium gas. Gas-dynamic virtual nozzles and extruder-based delivery approaches can be prepared and inserted into the endstation under anaerobic conditions (Calvey *et al.*, 2016 ▸, 2019 ▸; Schulz *et al.*, 2019 ▸; Knoška *et al.*, 2020 ▸). This setup enables the investigation of pre-di­oxy­gen binding at RT and, potentially, subsequent reaction steps initiated by controlled di­oxy­gen exposure. The drop-on-demand tape drive (Fuller *et al.*, 2017 ▸) can also be used to collect time-resolved data from di­oxy­gen dependent enzymes. Thus far, these approaches have not been adapted to enable anaerobic sample data collection at synchrotrons, where beam time is much more widely available and accessible to the majority of crystallographers than is presently the case for XFELs.

Since most synchrotron endstations are not equipped with an anaerobic sample chamber for data collection, a sample mount that maintains a localized anaerobic environment around the sample should provide a solution. Fixed-target serial crystallography mounts lend themselves to this because some, such as the fixed-target developed between I24, Diamond Light Source and Hamburg (Sherrell *et al.*, 2015 ▸), encase a solid support for the sample between two layers of film, which maintains crystal hydration during data-collection. We envisaged that these film layers could be adapted to create a sealed chamber capable of preventing di­oxy­gen entry at RT, enabling collection of anaerobic data at a synchrotron (or XFEL) light source.

**Figure 1:**
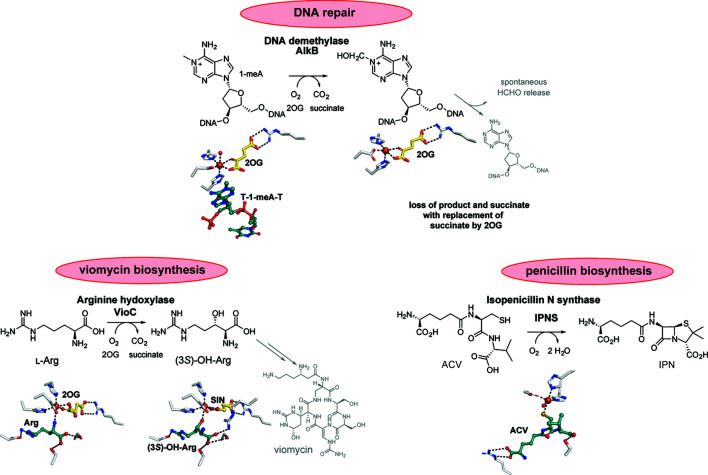
Summary of the reactions catalysed by the enzymes used in this study, *i.e.* the two 2OG-dependent oxygenases AlkB and VioC, and IPNS. The presence of a substrate in the active site is a clear indication of whether or not molecular dioxygen has diffused to the active site and reacted productively. The figure was constructed using the following structures: VioC:Fe:2OG:Arg (PDB entry 6y0n), VioC:Fe:SIN:(3*S*)-OH-Arg (PDB entry 6y12), AlkB:Fe:2OG:T-1-meA-T (PDB entry 6y0q), AlkB:Fe:2OG (PDB entry 6ypv) and IPNS:Fe:ACV (PDB entry 6y0o). SIN = succinate.

To explore the utility of the anaerobic fixed-target method, we used three models of di­oxy­gen-using enzymes (Fig. 1 ▸): two archetypal Fe(II) and 2-oxoglutarate dependent 2OG oxygen­ases (VioC and AlkB), and the structurally related oxidase IPNS.

(1) VioC (*Streptomyces vinaceus)* is a 2OG-dependent l-arginine hy­droxy­lase that catalyses the production of (3*S*)-hy­droxy­arginine from l-arginine and which is involved in the biosynthesis of tuberactin antibiotics (*e.g.* viomycin) which have been used to treat *Mycobacterium tuberculosis* infections (Helmetag *et al.*, 2009 ▸; Mitchell *et al.*, 2017 ▸; Holm *et al.*, 2019 ▸).

(2) AlkB (*Escherichia coli)* is a 2OG-dependent hy­droxy­l­ase that repairs alkyl­ation damage to DNA by hy­droxy­lating the removal of *N*-methyl groups on DNA to give a hemiaminal which fragments into the repaired DNA and formaldehyde (Falnes *et al.*, 2002 ▸; Trewick *et al.*, 2002 ▸; Aas *et al.*, 2003 ▸; Yi *et al.*, 2010 ▸; Yang *et al.*, 2008 ▸; Müller & Hausinger, 2015 ▸).

(3) IPNS (*Aspergillus nidulans)* is a non-heme Fe(II)-dependent oxidase that catalyses the biosynthesis of the precursor of all clinically relevant penicillin and cephalosporin antibiotics, *i.e.* isopenicillin N (IPN) by four-electron oxidation of its δ-(l)-α-amino­adipoyl-(l)-cysteinyl-(d)-valine tripeptide substrate (ACV) (Rabe *et al.*, 2018 ▸; Tamanaha *et al.*, 2016 ▸).

Following optimization, protein microcrystals of the aforementioned enzymes were prepared in an anaerobic chamber and co-crystallized with their respective substrates, Fe(II) and 2OG co-substrate, when appropriate. Since di­oxy­gen is required to trigger their reaction cycles, the presence of either substrate or product within the enzyme active site (Fig. 1 ▸) is a sensitive indicator of the absence or presence of di­oxy­gen at the active site. Hence, providing the structures can be solved at sufficiently high resolution, they serve as good test subjects for the anaerobic fixed-target method. As a control, we also collected data from air-exposed crystals, using the standard fixed-target chip setup (Sherrell *et al.*, 2015 ▸; Ebrahim, Appleby *et al.*, 2019 ▸). The results reveal the utility of the method, which enables routine serial data collection of di­oxy­gen dependent enzymes at synchrotron light sources.

## 2. Methods   

IPNS and AlkB were prepared as reported (Roach *et al.*, 1995 ▸; Woon *et al.*, 2012 ▸). VioC was prepared as described below. All recombinant proteins were >95% pure as judged by SDS–PAGE analysis.

### 2.1. Cloning of VioC in pNIC28   

Codon-optimized VioC (UniProt: Q6WZB0) from *S. vin­aceus* was cloned into the pNIC28-Bsa4 vector (Addgene, US) by ligation-independent cloning (LIC) (Savitsky *et al.*, 2010 ▸) using primers with an LIC overhang (Table S1 of the supporting information). A volume of 2 µl of the LIC-ready insert solution was mixed with 1 µl of LIC-ready vector at RT for 30 min, the ligation solution was then cooled on ice for 5 min and transformed into *Escherichia coli* XL10-Gold cells. Single colonies were picked and grown in 2YT medium; the plasmid was isolated using the GeneJET Plasmid Miniprep System (Thermo Scientific). The integrity of the insert was confirmed by DNA sequencing.

### 2.2. Purification of recombinant VioC   

*E. coli* T7 LysY cells containing the pNIC_VioC plasmid were grown in 2YT medium containing 50 µg ml^−1^ kanamycin; VioC production was induced with 0.5 m*M* iso­propyl β-d-thiogalactopyran­oside (IPTG) at an OD_600_ of 0.6. The cells were grown at 25°C overnight, then harvested by centrifugation (12 000*g*, 4°C); the resultant cell pellet was stored at −80°C. The frozen cell pellet (20 g) was resuspended in binding buffer (100 ml, 100 m*M* Tris, 20 m*M* imidazole pH 7.5), followed by the addition of DNase I and phenyl­methyl­sulfonyl fluoride (PMSF). Cells were lysed by sonication (9.9 s on, 9.9 s off, 5 min in total, 4°C, 60% amplitude, Sonics VibraCell VCX 500 with CV33 probe). The cell lysates were centrifuged (4°C, 12 000*g*, 30 min) then filtered (0.2 µm filter). The resultant mixture was loaded onto a HisTrap column (GE Healthcare, pre-equilibrated with binding buffer). Protein purification was performed by washing the column with binding buffer (20 CV) and elution of VioC using a gradient to 100% elution buffer (100 m*M* Tris pH 7.5, 250 m*M* imidazole, over 20 CV). The purity of the protein (>90%) was determined by SDS–PAGE analysis. The respective fractions were concentrated using a 10K centrifugal filter (Merck). The His-tag was cleaved using TEV protease at 4°C for 16 h. The cleaved protein was then purified using a second HisTrap column (GE Healthcare) using binding buffer for elution. Size-exclusion chromatography with a Superdex75 300 ml column (GE Healthcare) using size-exclusion buffer (20 m*M* Tris, 200 m*M* NaCl pH 7.5) resulted in highly purified recombinant VioC. The enzyme was concentrated to 20.7 mg ml^−1^, flash frozen in liquid nitro­gen and stored at −80°C.

### 2.3. Preparation of microcrystals   

All protein crystallizations were conducted within an anaerobic chamber maintained at 2 p.p.m. O_2_ or less (Belle Technologies, UK) with plates, solutions and other equipment used for crystallization allowed to degas within the chamber for at least 24 h (Roach *et al.*, 1996 ▸).

#### 2.3.1. VioC microcrystal preparation   

Seed crystals of VioC were prepared anaerobically in VDX plates (Hampton Research, US) as follows. Stock solutions of the recombinant enzyme, l-arginine, iron (II) ammonium sulfate and 2OG were transferred into the glove box 2 h before use. A crystallization screen was prepared with total well volumes of 500 µl, containing 0.05 *M* MgCl_2_ and varying the concentration of polyethyl­ene glycol (PEG) 550 [18–28%(*w*/*v*)] and the pH [0.1 *M* HEPES pH 7–8.5 (pH steps of 0.5)] along the horizontal and vertical axes, respectively. The purified VioC (12 mg ml^−1^ in 20 m*M* Tris pH 7.5, 200 m*M* NaCl) was incubated with 1.5 m*M* FeSO_4_, 1.5 m*M* l-arginine, 6 m*M* 2OG then mixed in a 1:1 ratio with the reservoir solution to give a final volume of 4 µl. Crystals were grown for 24–48 h until the longest dimension equalled approximately 150–200 µm; the crystals were then harvested and seeds prepared using a Seed Bead Kit (Hampton Research, US).

Anaerobic microcrystallization was performed using a batch method in 96-chimney well plates. In each well, 90 µl of precipitant [26%(*w*/*v*) PEG 550, 0.1 *M* HEPES pH 7.5, 0.05 *M* MgCl_2_] was mixed with 10 µl of protein solution (15 mg ml^−1^ VioC, 20 m*M* Tris pH 7.5, 200 m*M* NaCl, 2 m*M* FeSO_4_, 2 m*M* l-arginine, 4 m*M* 2OG) and 1 µl of seed stock. The plate was sealed and crystals were grown at RT for 24–48 h. The microcrystal slurry (15–20 µm average size) was then pooled by combining 10–15 wells in a 1.5 ml tube. Crystals were allowed to settle to the bottom of the tube; approximately 50% of the precipitant was then removed to give a final concentration of ∼5 × 10^6^ crystals ml^−1^ for data collection.

#### 2.3.2. AlkB microcrystal preparation   

Highly purified (>95% by SDS–PAGE) recombinant AlkB was prepared as described (Woon *et al.*, 2012 ▸) and stored in 50 m*M* Tris, pH 7.5, 100 m*M* NaCl, 1 m*M* DTT. AlkB (10.2 mg ml^−1^, 200 µl) was incubated with iron (II) ammonium sulfate (2.2 m*M*), 2OG (5.4 m*M*) and substrate (1.8 m*M* methyl­ated trinucleotide T-1-meA-T) in 50 m*M* Tris, pH 7.5 under anaerobic conditions at RT for 15 min prior to batch crystallization set-up. The resultant protein solution was mixed in a 1:5 ratio with precipitant solution [25%(*w*/*v*) PEG 3350, 5%(*v*/*v*) glycerol and 200 m*M* sodium formate, 1.6 ml] to give a final volume of 2 ml, and finally supplemented with 200 µl of seed stock solution. The crystallization solution was mixed carefully, then split into 40 wells of a 96-chimney well plates with a volume of 100 µl each, which were sealed (Polyolefin StarSeal, Starlab, UK) and stored without shaking. Microcrystals, with an average size of approximately 5 µm, appeared within 24 h. The wells were then combined and left to settle overnight. Before use, ∼80% of the supernatant was removed and the settled crystals were resuspended to give a concentrated microcrystalline slurry. An exact determination of microcrystal concentration was not possible.

#### 2.3.3. IPNS microcrystal preparation   

Highly purified (>95% by SDS–PAGE) recombinant IPNS was produced and purified as described and stored in 25 m*M* Tris, pH 8.0 (Roach *et al.*, 1996 ▸). IPNS crystals were grown anaerobically in 24-well hanging drop VDX plates (Hampton Research, US). The IPNS crystallization solution was produced by mixing 4 µl of freshly prepared 100 m*M* FeSO_4_ with 80 µl of 50–52 mg ml^−1^ IPNS, followed by a subsequent addition (4 × 5 µl) of ACV (2.0 mg in 20 µl 25 m*M* Tris pH 8.5). A screen, varying the pH (0.1 *M* Tris pH 8.1 to 8.7) and the salt concentration (Li_2_SO_4_ 1.5 to 2.0 *M*) was carried out. Crystals were prepared using the hanging-drop method by combining 3 µl of the reservoir and 3 µl of protein solutions. Needle-shaped crystals were used to prepare seeds using the Seed Bead Kit as described by the manufacturer (Hampton Research, USA).

Microcrystals were grown using the batch method; 6.5 µl of the IPNS–ACV–FeSO_4_ mixture (prepared as described above) was mixed with 90 µl of 1.7 *M* Li_2_SO_4_ and 0.1 *M* Tris pH 8.5. 1 µl of the seed stock was then added; the mixture was homogenized by pipetting then placed in a 96-chimney well plate (Corning, USA). The plate was sealed (Polyolefin StarSeal, Starlab, UK) and agitated using a microplate shaker (SciQuip, UK) at 700 r.p.m., until 40–60 µm crystals (longest dimension) had grown. Microcrystal growth usually took between 16 and 30 h and appeared to be dependent upon the temperature and the humidity of the anaerobic chamber. A final concentration of ∼2 × 10^7^ crystals ml^−1^ was typically achieved.

### 2.4. Oxygen-sensitive dye spectroscopy   

**Figure 2:**
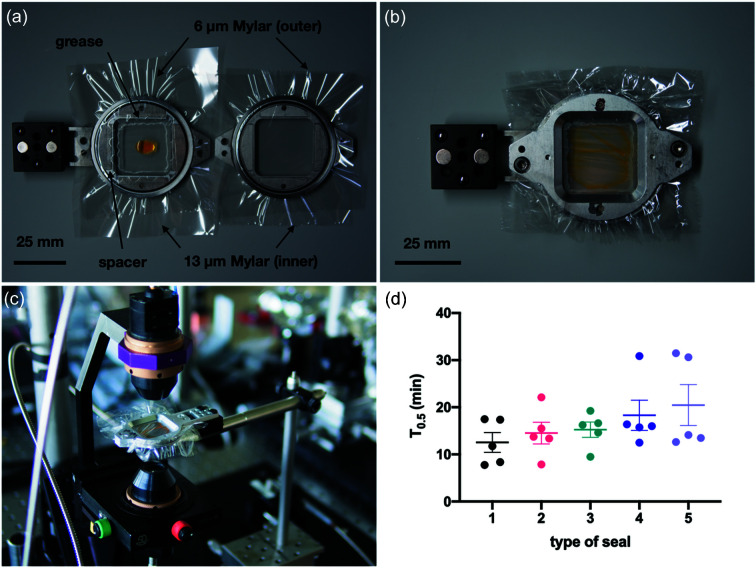
Overview on the ‘sheet-on-sheet’ setup and results from the fluorescence measurements. (*a*)–(*c*) show how the film sandwiches were prepared and the fluorescence recorded using a micro-spectrometer. This particular case shown used a 3.4 × 3.4 cm spacer with vacuum grease and two pieces of 6 and 13 µm Mylar. (*d*) Fluorescent half-lives manifested with the five different types of seal: (1) 2.5 × 2.5 cm spacer + 13 µm Mylar, (2) grease + 13 µm Mylar, (3) 2.5 × 2.5 cm spacer + grease + 13 µm Mylar, (4) 3.4 × 3.4 cm spacer + 13 µm Mylar, and (5) 3.4 × 3.4 cm spacer + grease + 13 and 6 µm Mylar. Single measurements are shown as full circles. Mean values and the standard deviations are represented by bars and whiskers, respectively.

The presence of di­oxy­gen was inferred by altered fluorescence of the dye Tris(4,7-di­phenyl-1,10-phenanthroline) ruthenium bis­(hexa­fluoro­phosphate): TBF (Carbosynth, UK). TBF is a luminescent dye with excitation and emission maxima of 450 and 615 nm, respectively (Bolink *et al.*, 2006 ▸). TBF is fluorescent under anaerobic conditions, but fluorescence is quenched by di­oxy­gen (Bacon & Demas, 1987 ▸; Klimant & Wolfbeis, 1995 ▸). Since the change in fluorescence of TBF is non-linear, care was taken to ensure that the concentration of di­oxy­gen inside the chamber while loading the dye remained below 100 p.p.m. Differences in the starting concentration of di­oxy­gen were thus minimized and errors associated with this non-linearity were consequently reduced. TBF was dissolved to a final concentration of 0.625 m*M* in a solution of 50%(*v*/*v*) ethanol and 25%(*v*/*v*) PEG 3350 and degassed by placing the solution in an anaerobic chamber for at least 24 h. 20 ul of the dye solution was pipetted onto fixed-target holders (Mehrabi *et al.*, 2020 ▸) that were pre-assembled with Mylar films of different thickness: 13 µm (Goodfellow, UK) and 6 µm (SPEX Certiprep, UK), nuclear grade grease (Superlube, USA) and adhesive inter-seal (4titude, UK) combinations (Fig. 2 ▸). Subsequently, the holders were assembled and transferred immediately from the anaerobic chamber to the spectrometer.

Spectroscopy measurements employed an Andor 303i Shamrock spectrometer (Oxford Instruments, UK) using a deuterium–halogen light source (Ocean Optics, UK). Light was coupled from the light source to the spectrometer using 600 µm-diameter optical fibres (Ocean Optics), passed through a 500 nm shortpass optical filter (FES0500, Thorlabs, Germany) and focused to a spot of approximately 50 µm at the sample position. Emitted light was collected at 180° from the excitation source and coupled to the spectrometer through a 600 nm longpass filter (FEL0600, Thorlabs). Optical spectra were taken as a kinetic series for 4 h. Each measurement was a summation of 1000 single 4.45 ms exposure spectra [470 to 750 nm, Fig. S1(*a*) of the supporting information]. Accumulated spectra were smoothed and the changes in the fluorescence signal were plotted as the absolute difference between the number of counts at 615 nm with 550 nm used as a reference [Fig. S1(*b*)]. Datapoints were fitted to a single exponential decay model, which enabled calculation of half-lives of the dye fluorescence for each particular setup. All signal-processing was achieved using *Origin* (Pro) (version 2015, OriginLab Corporation, USA) and plotted with *Origin* or *Prism 8* (GraphPad).

### 2.5. Anaerobic chipless chip preparation for data collection   

Fixed-target chip holders (Mehrabi *et al.*, 2020 ▸) were used in a ‘sheet-on-sheet’ format (Axford *et al.*, 2016 ▸; Doak *et al.*, 2018 ▸). Mylar film (13 and 6 µm thickness), nuclear grade grease and 50 µm-thick double-sided adhesive tape were used in the construction of the anaerobic holders and degassed prior to the experiment in an anaerobic chamber (Belle Technologies, UK) located in the Research Complex at Harwell, 30 m from Diamond Light Source. The vacuum grease was degassed for at least 24 h followed by storage in the anaerobic chamber for times ranging from two days to weeks before use; the Mylar was stored in the chamber for as long as reasonably possible (at least 2 h).

The Mylar sandwiches (Fig. 2 ▸) were constructed from four sheets: two of 13 µm and two of 6 µm. Each side of the holder contained one 6 µm and one 13 µm sheet, with the 13 µm sheet closest to the sample. Once the 6 and 13 µm Mylar sheets were secured on each side of the holder, a square piece of 50 µm double-sided adhesive film was attached to the edge of the holder. Further towards the centre of the holder from the adhesive and around the sample position, a ring of vacuum grease was extruded from a plastic syringe through a 200 µl pipette tip. 15 µl of the microcrystalline slurry was then pipetted into the centre of the Mylar sandwich so that it was surrounded by the ring of grease and the adhesive film. The two sides of the holder were then placed together and a good seal was made between each side of the Mylar sandwich and the vacuum grease ring and the adhesive film. The surface tension of the sample solution typically suspended the sample in the centre of the films with a sample thickness of at least 50 µm and an area of >4 mm^2^. The holder was then placed inside two airtight storage containers (Lock & Lock) chosen in part for ease and speed of use, transferred to the beamline as quickly as possible, and data collection was started. The whole transport process from leaving the anaerobic chamber to the beginning of data collection took ∼2 min.

### 2.6. Data collection   

All data were collected on beamline I24 at Diamond Light Source using a beam size of 8 × 8 µm (FWHM) and 12.8 keV energy X-rays using the fixed-target instrumentation described in the work by Owen *et al.* (2017 ▸). All crystals were exposed to the beam for 10 ms using an incident flux of 3.0 × 10^12^ photons s^−1^.

#### 2.6.1. O_2_-exposed samples   

Data for the di­oxy­gen-exposed structures were collected from protein crystals loaded onto silicon wafers or ‘chips’ containing 25 600 apertures (Sherrell *et al.*, 2015 ▸; Ebrahim, Appleby *et al.*, 2019 ▸). In brief, to prepare each chip, the microcrystalline slurry was pipetted onto the well side of the chip and the excess buffer was removed by applying a vacuum to the other side. The chip was then placed in an aluminium holder that encloses the chip within two sheets of 6 µm Mylar (Mehrabi *et al.*, 2020 ▸). This entire process was conducted inside a tent where the humidity was maintained at >85%. Once the chip had been encased with the holder, it was removed from the humidity tent and transferred to the beamline.

#### 2.6.2. Anaerobic samples   

Anaerobic data were collected from the sandwiches using the same fixed-target approach, though with a custom step size to produce a series of still diffraction patterns from crystals randomly positioned on the film. The sample was mounted on the beamline and the beam was aligned to a corner of the sample sandwich. Data collection was immediately initiated and the sample was rastered through the X-ray beam in a regular snake-like pattern. The images were collected from a 2.5 × 2.5 mm (or 2.5 × 5.0 mm) area of the sample with 25 µm spacing between the shots. Each collection yielded 10 000 (20 000) images and took less than 5 min, including the time taken to align the sample, hutch search, and for the data collection to begin and complete.

### 2.7. Data processing, model building and refinement   

Diffraction images were processed using *dials.stills_process* (Winter *et al.*, 2018 ▸). After an initial round of spot-finding, indexing and integration, a round of metrology refinement was conducted. Using the detector parameters, a second round of *stills_process* (Brewster *et al.*, 2018 ▸) was then performed. Data merging was initially performed using *PRIME* (Hattne *et al.*, 2014 ▸; Sauter *et al.*, 2014 ▸; Sauter, 2015 ▸; Uervirojnangkoorn *et al.*, 2015 ▸) followed by molecular replacement with *PHASER* (McCoy *et al.*, 2007 ▸). This provided a reference model with a compatible unit cell used by the *cxi.merge* component of *cctbx.xfel* in final merging (Young *et al.*, 2016 ▸). The resolution cut-off for the merged data was determined based on a combination of two criteria: multiplicity in the highest resolution shell (around tenfold) and CC_1/2_ (uniform decrease) (Fuller *et al.*, 2017 ▸).

Structures were solved by isomorphous molecular replacement using reported structural datafiles of VioC (PDB entry 6alm, Mitchell *et al.*, 2017 ▸), AlkB (PDB entry 2fd8, Yu *et al.*, 2006 ▸) and IPNS (PDB entry 1blz, Roach *et al.*, 1997 ▸) as search models. All five structures were iteratively fitted and refined using *PHENIX* (Adams *et al.*, 2002 ▸) and *Coot* (Emsley *et al.*, 2010 ▸). Processing and refinement statistics for VioC and AlkB anaerobically and O_2_-exposed, and IPNS anaerobically are found in Table S2.

## 3. Results   

### 3.1. Developing an anaerobic fixed target   

#### 3.1.1. Fixed-target di­oxy­gen permeability   

The use of a fixed-target approach enables high-throughput serial data collection with high hit rates and low sample consumption (Sherrell *et al.*, 2015 ▸; Owen *et al.*, 2017 ▸). For some types of samples, the requirement to maintain a strictly anaerobic environment during data collection creates several challenges. Initial attempts, which utilized the standard silicon nitride chip setup (Sherrell *et al.*, 2015 ▸), failed as this assembly proved ineffective at preventing air diffusion into the microcrystalline sample. We therefore worked to develop an alternative setup that is easier to use and which minimizes gas permeability to the sample.

Different setups were assessed by monitoring the reduction in fluorescence intensity of a di­oxy­gen-sensitive dye in response to air exposure. For practical reasons, we performed experiments with standard chip holders in a ‘sheet-on-sheet’ format, wherein the dye solution was directly sandwiched between Mylar foils (Doak *et al.*, 2018 ▸). The results reveal that use of vacuum grease and a 50 µm adhesive inter-seal of different sizes (2.5 × 2.5 cm or 3.4 × 3.4 cm) reduces gas permeation [up to ∼*t*_0.5_ = 30 min, see Fig. 2 ▸ (*t*_0.5_ is defined here as the time taken for half the fluorescence to decay)]. Due to the fact that the most promising setup (6 and 13 µm Mylar foils secured on either side of the holder, surrounded by an 3.4 × 3.4 cm inter-seal and grease) could not accommodate silicon chips, in this work we pursued a ‘chipless chip’ approach (Doak *et al.*, 2018 ▸) in which 15 µl of the microcrystalline slurry was directly sandwiched between the Mylar foils. We note that the limitation of silicon chip and multi-layer film compatibility could be addressed *via* a small modification to the chip holder design, which will be pursued in future work. The adaptation of the ‘sheet-on-sheet’ approach to anaerobic samples enabled us to obtain high-resolution structures of substrate complexes of di­oxy­gen-using enzymes, as described below.

#### 3.1.2. Comparison of the anaerobic and O_2_-exposed strategies   

**Figure 3:**
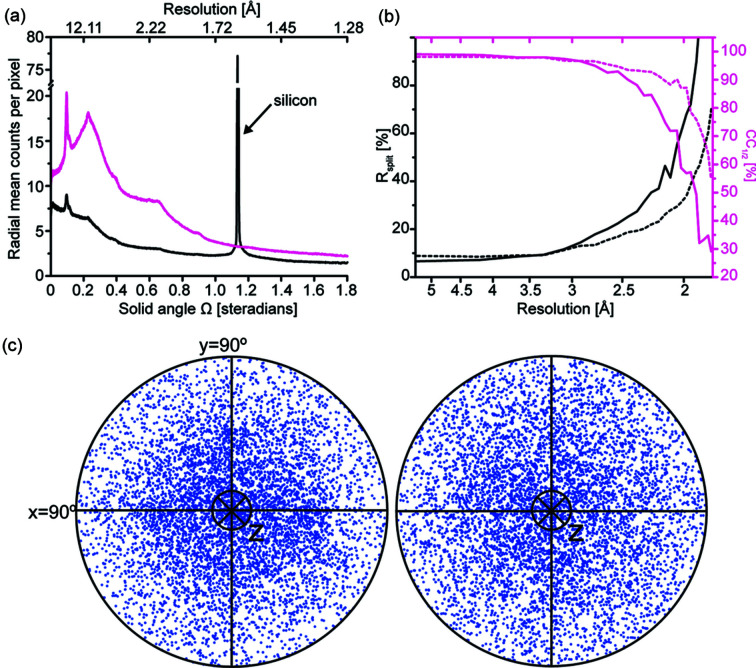
Analysis of VioC data. (*a*) Normalized average background count for anaerobic (magenta) and air-exposed setups (black) resulting from ∼3.0 × 10^10^ photons at 12.8 keV with a beam size of 8 × 8 µm. (*b*) *R*_split_ (black) and CC_1/2_ (magenta) plotted against the resolution for anaerobic VioC:Fe:2OG (solid line) and O_2_-exposed VioC:Fe:succinate (dashed line) datasets. The analysis was done for 10 200 randomly selected integrated frames. (*c*) Stereographic projections illustrating the crystal orientation of 6400 randomly selected crystals for air-exposed VioC:Fe:2OG (left) and anaerobic VioC:Fe:succinate (right) datasets. The plots represent the direction of the *hkl* 001 of each crystal relative to the beam direction (*z*). For data collected on chips, the direction of *hkl* 001 to <45° movement away from the beam direction seems to be favoured.

Although the sheet-on-sheet approach simplifies sample mounting, enables visualization and is less likely to damage the crystals than the standard chip-based method (Doak *et al.*, 2018 ▸), it has some disadvantages. In particular, we were concerned about the potential for increased background scatter, preferred orientations and decreased hit-rates. To assess the contribution of the additional Mylar (two additional 13 µm Mylar foils) and the surrounding mother liquor to the X-ray scattering background, we compared the merging statistics for a fixed number of integrated frames, which were recorded from the same sample using two different setups as a measure of data quality. The results in Fig. 3 ▸ show the comparison using VioC microcrystals, for which data were recorded for the same batch of microcrystals. Compared with the chip-based data, the ‘sheet-on-sheet’ data manifest a higher background level across the entire resolution range [Fig. 3 ▸(*a*)]. The better quality data recorded on chips is reflected in the lower overall *R*_split_ (17.6 and 14.0% for the anaerobic and O_2_-exposed datasets, respectively) and slower falloff of CC_1/2_ values [Fig. 3 ▸(*b*)]. Although the comparison shows that the multi-film sheet-on-sheet approach is worse than the chip-based approach from this perspective (at least in this case), the difference is small. The impact on merging resolution, at least for the microcrystals and energies used, is also small (*N*_obs_ ≃ 10 at 1.89 and 1.78 Å for the ‘sheet-on-sheet’ and chip-based data, respectively), with other factors, such as crystal quality or the number of integrated frames, likely having a greater effect on the overall data quality.

**Figure 4:**
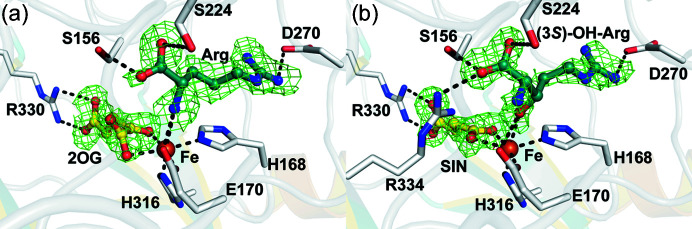
Comparison of the anaerobic and O_2_-exposed VioC structures. Close-up views of (*a*) a Polder omit map to 1.86 Å resolution of the active site of the VioC:Fe:2OG:Arg substrate complex structure reported in this study (PDB entry 6y0n) displayed at 2.0σ contour level, carved around the omitted arginine and 2OG, and (*b*) a Polder omit map of the active site of the VioC:Fe:2OG:(3S)-OH-Arg complex reported in this study (PDB entry 6y12) displayed at 3.0σ contour level, carved around the omitted (3S)-OH-Arg product and succinate highlighting the coordination of the substrate and product of VioC *via* D270, E170, Fe, S224, S156 and the conformationally flexible residue R334, which is solely present in the (3S)-OH-Arg product complex and disordered in the VioC:Fe:2OG:Arg substrate complex (Mitchell *et al.*, 2017 ▸). (3S)-OH-Arg = (3S)-hy­droxy-l-Arg; SIN = succinate.

Using both the chip and sheet-on-sheet approaches, we were able to obtain good quality, complete datasets in low-symmetry space groups for AlkB and VioC microcrystals (*P*1 and *C*2; Figs. 4 ▸ and 5 ▸). The sheet-on-sheet method offered some additional benefits compared with microcrystals loaded onto a chip using the weak-vacuum loading technique (Oghbaey *et al.*, 2016 ▸). The distribution of crystal orientations within the sheets was more varied [Fig. 3 ▸(*c*)], possibly because the crystals are freer to lie on any face rather than becoming wedged in chip apertures (Davy *et al.*, 2019 ▸). Furthermore, a smaller volume of microcrystalline slurry was required to collect a complete dataset with the sheet-on-sheet method. Given the average integration ratio of the data collected using the sheet-on-sheet and chip-based methods (7 versus 17% for VioC, respectively) and the difference in the volume loaded (15 versus 150 µl, respectively), comparable integration ratios were achieved with approximately fourfold less material using the sheet-on-sheet approach. The sheet-on-sheet method was also better suited to samples with particularly small crystals, such as AlkB, for which the ∼5 µm crystals were typically too small to load onto the chips with a minimum aperture size of 7 µm. The overall difference in efficiency was even more substantial for AlkB, since the average integration rate for the sheet-on-sheet setup was almost 100 times more efficient than for the chip-based setup (15 versus 1.6%, respectively). It should be noted, however, that new loading technologies, such as on-demand acoustic droplet ejection (Roessler *et al.*, 2016 ▸) or piezoelectric loading strategies can also reduce sample consumption for chip-based data collection (Davy *et al.*, 2019 ▸). Unfortunately, those on-demand sample loading methods also require more equipment and are much more complex than the sheet-on-sheet methods described here.

### 3.2. Oxygen dependent enzyme structures solved using anaerobic fixed-target methods   

#### 3.2.1. VioC microcrystal structures   

We solved RT structures of VioC which were crystallized in the presence of Fe(II), 2OG and arginine, both in the absence of di­oxy­gen and following 1 h exposure to air (at 1.9 and 1.7 Å, respectively) using the sheet-on-sheet fixed-target method described above. The overall structures are very similar to each other (main chain RMSD: 0.223 Å between PDB entry 6y0n and PDB entry 6y12) and those reported for VioC (Helmetag *et al.*, 2009 ▸; Mitchell *et al.*, 2017 ▸). Importantly, clear differences in the active sites of the two structures were observed, with apparently complete conversion of 2OG to succinate and carbon dioxide (not observed), and of arginine to (3*S*)-hydroxy­arginine in the air-exposed structure. This observation demonstrates that both the VioC crystals are catalytically active and the utility of the sheet-on-sheet method for determining ground-state structures of either the anaerobic substrate or product complexes. Both the structures manifest the 2-His-1-carboxyl­ate facial triad involved in iron coordination (His168, His316 and Glu170, Fig. 4 ▸). As previously reported for VioC and related 2OG oxygenase structures, *e.g.* clavaminic acid synthasec (CAS) and l-asparagine oxygenase (AsnO) (Strieker *et al.*, 2007 ▸; Zhang *et al.*, 2002 ▸), the 2OG ligates the Fe in a bidentate manner *via* its oxalyl group and the C1 carboxyl­ates of 2OG in the anaerobic VioC:Fe:­2OG:Arg complex (PDB entry 6y0n) being coordinated *trans* to the proximal histidine (His168) of the triad; succinate is bound in a monodentate manner, also *trans* to His168. The non-metal-ion ligating co-substrate/co-product carboxyl­ate is positioned to interact with the guanidinium group of Arg330 *via* a salt bridge. Binding of both the arginine substrate and the 3-hy­droxy­arginine product in VioC is characterized by interactions with Asp270 (with the substrate/product guanidino group), Ser224 and Ser156 (with the substrate/product carboxyl­ate group), and *via* a hydrogen bond between Glu170 (part of the 2-His-1-carboxyl­ate triad) and the α-amino group of the substrate/product. In the di­oxy­gen exposed structure, however, the (3*S*)-OH-Arg is positioned to coordinate Fe *via* its (3*S*)-hydroxyl group. Structures of VioC in complex with arginine solved under cryo-conditions have revealed a second conformation of the substrate (Helmetag *et al.*, 2009 ▸; Mitchell *et al.*, 2017 ▸), which we did not observe in our structure, probably due to its lower resolution. An overview of VioC structures and superimposition of our structures with those reported in the literature is given in Fig. S2.

#### 3.2.2. AlkB microcrystal structures   

**Figure 5:**
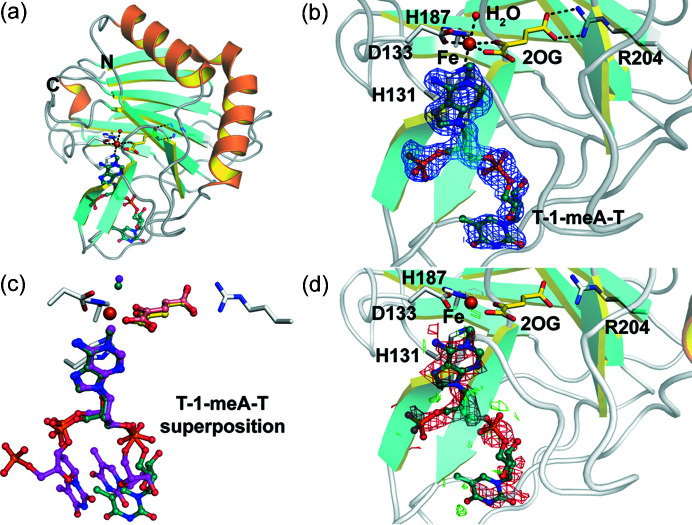
Comparison of the anaerobic and O_2_-exposed AlkB structures. (*a*) Structural overview of the anaerobic AlkB:Fe:2OG:T-1-meA-T complex structure (PDB entry 6y0q, this study). (*b*) Composite omit map to 1.75 Å resolution of the active site carved around the methyl­ated DNA fragment displayed at 1.0σ contour level. (*c*) Superimposition of the cryogenic AlkB:Fe:2OG:T-1-meA-T complex [magenta and salmon, PDB entry 2fd8 (Yu *et al.*, 2006 ▸)] and the serial RT structure reported here (cyan and yellow, PDB entry 6y0q). (*d*) *F*_obs_ − *F*_obs_ isomorphous difference maps carved around the methyl­ated DNA fragment contoured at +2.0σ (green) and −2.0σ (red) for the AlkB:Fe:2OG structure [PDB entry 6ypv (this study)] relative to the AlkB:Fe:2OG:T-1-meA-T structure (PDB entry 6y0q).

Diffraction data on the second model non-heme Fe-2OG dependent di­oxy­genase, AlkB, were also collected from microcrystals prepared both anaerobically and after exposure to air (structures solved to 1.9 and 1.7 Å resolution, respectively, see Fig. 5 ▸). As anticipated, in the AlkB:Fe:2OG:T-1-meA-T substrate complex [Figs. 5 ▸(*a*) and 5(*b*)], the iron is coordinated through the AlkB 2-His-1-carboxyl­ate motif (residues His131, Asp133 and His187) by bidentate chelation of 2OG, with the 2OG C5 carboxyl­ate forming a salt bridge with the guanidinium group of Arg204, as reported (*e.g.* PDB entry 2fd8) in the work by Yu *et al.* (2006 ▸). However, whereas the conformation of the *N*-methyl­ated adenine of the trinucleotide substrate T-1-meA-T is superimposable with that in the reported structure, there are clear differences in the conformations of the neighbouring thymine nucleotides [Fig. 5 ▸(*c*)]. This is likely to be a result of the differences in crystal packing (this study: *P*1, *a* = 36.4, *b* = 39.1, *c* = 40.8 Å, α = 79.0, β = 78.0, γ = 66.9° compared with the reported PDB entry 2fd8: *P*4_3_, *a* = 40.7, *b* = 40.7, *c* = 118.3 Å; α = β = γ = 90°; main-chain RMSD = 0.495 Å between PDB entry 6y0q and PDB entry 2fd8). Although the air-exposed VioC crystals reveal a ‘trapped’ (3*S*)-OH-Arg product complex, exposure of the AlkB microcrystals to air for the same time period (1 h) results in an AlkB:Fe:2OG complex wherein the de­methyl­ated DNA product ligand has dissociated and/or is disordered. This is illustrated *via* comparison of *F*_obs_ − *F*_obs_ isomorphous difference maps for the AlkB:Fe:2OG (PDB entry 6ypv) and AlkB:Fe:2OG:T-1meA-T (PDB entry 6y0q) structures which exhibit strong negative difference electron density over the DNA ligand [Fig. 5 ▸(*d*)]. This interpretation of the maps is consistent with the relative stoichiometries for the 2OG and T-1meA-T ligands (∼3:1) used under anaerobic crystallization conditions. The overall folds for the AlkB:Fe:2OG (PDB entry 6ypv) and the AlkB:Fe:2OG:T-1-meA-T (PDB entry 6y0q) structures are very similar (main-chain RMSD: 0.234 Å). The observed product/succinate release for microcrystalline AlkB catalysis is in line with reported observations where the proposed hemiaminal intermediate product complex could only be observed crystallographically after chemical cross-linking of the substrate to the enzyme (Yang *et al.*, 2008 ▸). Substitution of the succinate co-product for 2OG in the *in crystallo* catalysis is likely to be observed in part due to the excess of 2OG in solution and higher affinity of AlkB for 2OG compared with succinate (Bleijlevens *et al.*, 2008 ▸).

#### 3.2.3. IPNS microcrystal structure   

There are several reports on structures of anaerobic and O_2_-exposed IPNS:Fe:ACV complexes including high-pressure di­oxy­gen experiments using macrocrystals (Rutledge *et al.*, 2002 ▸; Burzlaff *et al.*, 1999 ▸). The IPNS:Fe:ACV complex reacts efficiently with di­oxy­gen, with turnover of IPNS:Fe:ACV crystals observed at >2 p.p.m. atmospheric di­oxy­gen (data not shown); IPNS thus serves as an excellent model system to validate the anaerobic fixed-target approach. All previously reported structures of IPNS deposited in the PDB to date (>35) have been derived from crystal forms with hexagonal [*e.g.* PDB entry 1oc1 (Long *et al.*, 2003 ▸); *P*3_1_21, *a* = *b* = 101.0, *c* = 115.7 Å] or plate (*e.g.* PDB entry 1bk0; *P*2_1_2_1_2_1_, *a* = 46.8, *b* = 71.5, *c* = 101.0 Å) morphologies. Notably, following optimization our work employed microcrystalline IPNS:Fe:ACV with a needle morphology (PDB entry 6y0o; *P*2_1_2_1_2_1_, *a* = 41.9, *b* = 75.8, *c* = 102.0 Å) and these anaerobic enzyme–substrate crystals are active upon exposure to di­oxy­gen as demonstrated in several RT studies, which will be reported in future work.

**Figure 6:**
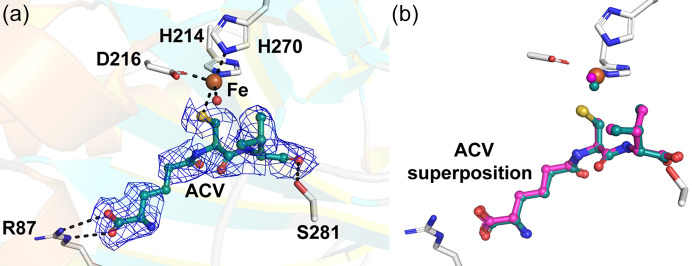
Structural comparison of anaerobic IPNS structures collected with RT serial fixed-target methods and under cryogenic conditions. (*a*) Composite omit map of IPNS:Fe:ACV displayed at 1.0σ contour level to 2.2 Å resolution, carved around the ACV substrate (PDB entry 6y0o). The ACV and residues R87, S281 and the Fe are shown as balls and sticks. (*b*) Superimposition of the cryogenic (magenta, PDB entry 1bk0) and serial RT (cyan, PDB entry 6y0o, this study) IPNS:Fe:ACV complex structures. The comparison reveals no differences in the conformation of the ACV substrate, with minor differences in the position of the metal-coordinating water.

The anaerobic IPNS structure determined clearly shows the ‘2-His-1-carboxyl­ate’ triad of the metal-ligating residues [His214, Asp216 and His270 (Fig. 6 ▸)]; the coordination sphere of Fe is completed by a water molecule and the cysteinyl sulfur of the substrate ACV. Importantly, there was no evidence for turnover to give the IPN product within the active site (as we have observed in ongoing studies), validating the anaerobic nature of the microcrystallization and data collection. Superimposition of the RT anaerobic structure reported here and the analogous reported cryogenic structure (Roach *et al.*, 1997 ▸) shows no substantial differences in the overall structure [main-chain RMSD = 0.346 Å between PDB entry 6y0o (this study) and PDB entry 1bk0] and within the active site [Fig. 6 ▸(*b*)]. One difference occurs in the apparent position of the Fe-coordinating water, though this might in part reflect the limited resolution of the fixed-target structure (resolution of 2.2 versus 1.3 Å for the fixed-target and cryogenic structures, respectively).

## 4. Discussion   

Routine data collection of cryogenic samples is an ubiquitous tool of crystallographic structure determination at synchrotrons; the holding of samples at 100 K both reduces the impact(s) of radiation-induced alterations and, in the case of anaerobic samples, prohibits the diffusion of di­oxy­gen. Within the field of serial synchrotron RT data collection, equivalent tools for anaerobic data collection have been lacking. Our work describes the first RT fixed-target method suitable for data collection from anaerobic samples at both synchrotron and XFEL light sources.

The method is an adaptation of the ‘sheet-on-sheet’ approach (Doak *et al.*, 2018 ▸), enabling gentle handling of microcrystals and ensures modest sample consumption and is easy to execute in a glovebox. Since the microcrystals are not localized in fixed apertures, they enjoy a greater degree of rotational freedom and, therefore, random orientations in reciprocal space. As the microcrystals are not in fixed positions, experiments which focus on the iterative investigation of the same crystal (*e.g.* dose series or pump–probe experiments) may not be feasible. Another potential disadvantage of the new method compared with the standard chip setup (Sherrell *et al.*, 2015 ▸; Ebrahim, Appleby *et al.*, 2019 ▸) is the higher background noise generated from the additional Mylar sheets and larger amount of solution surrounding the crystals. In the cases of the protein microcrystals studied here, the deleterious impact was small, but it could be problematic for data collection with very small crystals or when using low X-ray energies. As demonstrated by the lack of turnover observed with two 2OG oxygenases and IPNS, our method slows di­oxy­gen penetration to a level that enables data collection within a reasonable timeframe. Our fluorescence results clearly show di­oxy­gen diffusion through the films does occur with significant variations in half-life which may reflect the experimenters’ experience level and also differences in the quality of the holder seal. These variations underline the importance of careful evaluation of the recorded data, especially if no *in situ* experimental validation of the oxidation state is in place.

Studies on the application of the method to investigations on the mechanisms of 2OG oxygenases and IPNS will be reported in due course. Future work will focus on developing mounts such that the existing fixed targets can be mounted inside the holder. This should better allow pump–probe experiments with either X-rays or an optical laser. Work is also ongoing to improve on the seal repeatability and extend the ‘time window’ for maintaining the anaerobic conditions of the sample. This includes use of materials other than Mylar that have both a reduced di­oxy­gen permeability and a reduced X-ray scattering background. A key future development will be the incorporation of *in situ* spectroscopic verification of the sample oxidation state. This could be achieved either by exploiting a di­oxy­gen-sensitive probe added to the crystals or by the distinct spectral signatures of inherent metals bound in the protein active centres.

## 5. Related literature   

The following reference is cited in the supporting information: Bury *et al.* (2018 ▸).

## Supplementary Material

Supporting tables and figures. DOI: 10.1107/S2052252520010374/jt5050sup1.pdf

PDB reference: VioC:Fe:SIN:(3S)-OH-Arg, 6y12

PDB reference: IPNS:Fe:ACV, 6y0o

PDB reference: AlkB:Fe:2OG, 6ypv

PDB reference: VioC:Fe:2OG:Arg, 6y0n

PDB reference: AlkB:Fe:2OG:T-1-meA-T, 6y0q
