# Supplementary material for: Anaerobic fixed-target serial crystallography
Source: IUCrJ. 2020 Aug 21;7(Pt 5):901–12. doi: 10.1107/S2052252520010374 (PMC7467159; doi:10.1107/S2052252520010374)
Supplement: Supplementary file 1 [file m-07-00901-sup1.pdf]

IUCrJ

Volume 7 (2020)

Supporting information for article:

Anaerobic fixed-target serial crystallography

Patrick Rabe, John H. Beale, Agata Butryn, Pierre Aller, Anna Dirr, Pauline A. Lang, Danny N. Axford, Stephen B. Carr, Thomas M. Leissing, Micheal A. McDonough, Bradley Davy, Ali Ebrahim, Julien Orlans, Selina L. S. Storm, Allen M. Orville, Christopher J. Schofield and Robin L Owen

Table S1 LIC primers used in the cloning of the codon-optimised VioC into pNIC28-Bsa4 vector.

Oligo name	Sequence (5´-3´)	Tm (°C)
LIC_VioC fw	TACTTCCAATCCATGATGACCGAAAGT CCGACCACACATCATGG	85.5
LIC_VioC rev	TATCCACCTTTACTGGCGCTGACCCAG AACACGGC	83.3

Table S2 Data collection and refinement statistics. Absorbed doses were calculated using RADDOSE-3D (Bury *et al.*, 2018).

**Datasets**	**VioC:Fe:2OG: Arg** (PDB ID: 6Y0N)	**VioC:Fe:SIN: (3*S*)-OH-Arg** (PDB ID: 6Y12)	**AlkB:Fe:2OG: T-1meA-T**(PDB ID: 6Y0Q)	**AlkB:Fe:2OG** (PDB ID: 6YPV)	**IPNS:Fe:ACV** (PDB ID: 6Y0O)
**Data Collection**					
Beamline (Wavelength, Å) and detector	DLS I24 (0.9686), Pilatus3 6M				
Data Processing	DIALS (integration), cctbx.xfel (merging)				
Diffraction weighted dose / kGy	36	36	68	68	38
Number of images used	12318	16594	11778	4640	9449
Space group	C 2	C 2	P 1	P1	$P2_12_12_1$
**Cell dimensions**					
a, *b*, *c* (Å)	82.0, 66.6, 63.6	82.4, 67.1, 64.0	36.4, 39.1, 40.8	37.0, 39.1, 40.9	41.9, 75.8, 102.0
α, β, γ (°)	90, 110.2, 90	90, 110.2, 90	79.0, 78.0, 66.9	78.1, 76.0, 66.5	90, 90, 90
No. of	1	1	1	1	1

molecules/ASU					
No. reflections	27077 (2700)	36065 (3574)	20346 (1028)	11994 (593)	16880 (600)
Resolution (Å)	59.69-1.86 (1.93-1.86)	50.69-1.70(1.76-1.70)	35.67-1.75 (1.81-1.75)	39.35-2.10 (2.13-2.10)	60.85-2.20 (2.24-2.20)
R_{split}	0.232 (0.820)	0.215 (0.841)	0.178 (0.789)	0.333 (0.617)	0.235 (1.004)
<I>	74992 (6733)	59601 (4048)	6663 (660)	11298 (1965)	65916 (16988)
<I/σ(I)>	28.8 (2.3)	34.2 (2.3)	37.2 (2.8)	48.4 (8.795)	15.0 (1.4)
CC-1/2	0.94 (0.28)	0.95 (0.26)	0.98 (0.26)	0.87 (0.41)	0.96 (0.18)
Completeness (%)	100 (100)	99.9 (99.4)	100 (100)	100 (99.9)	97.7 (99.8)
Multiplicity	86.89 (10.86)	62.41(10.54)	57.57 (10.60)	25.8 (12.92)	130.22 (7.96)
Wilson B value ($Å^2$)	22.73	19.93	18.39	23.04	24.74
**Refinement**	PHENIX				
R_{work}/R_{free}	0.1886/0.2283	0.1819 /0.2103	0.1622/0.1914	0.2195/0.2506	0.2088/0.2474
No. atoms	2677	2787	1738	1593	2761
- Enzyme	2515	2574	1548	1510	2614
- Ligand	11	9	54	11	36
- Water	151	204	136	72	112
Average B-factors	33.26	26.87	24.24	26.83	30.09
- Enzyme	32.91	26.08	22.75	26.44	29.94
- Ligand	53.12	23.44	38.27	33.70	40.68
- Water	37.73	36.93	35.65	33.98	30.21
R.m.s deviations					
- Bond lengths (Å)	0.003	0.004	0.004	0.004	0.002
- Bond angles (°)	0.62	0.71	0.68	0.54	0.49

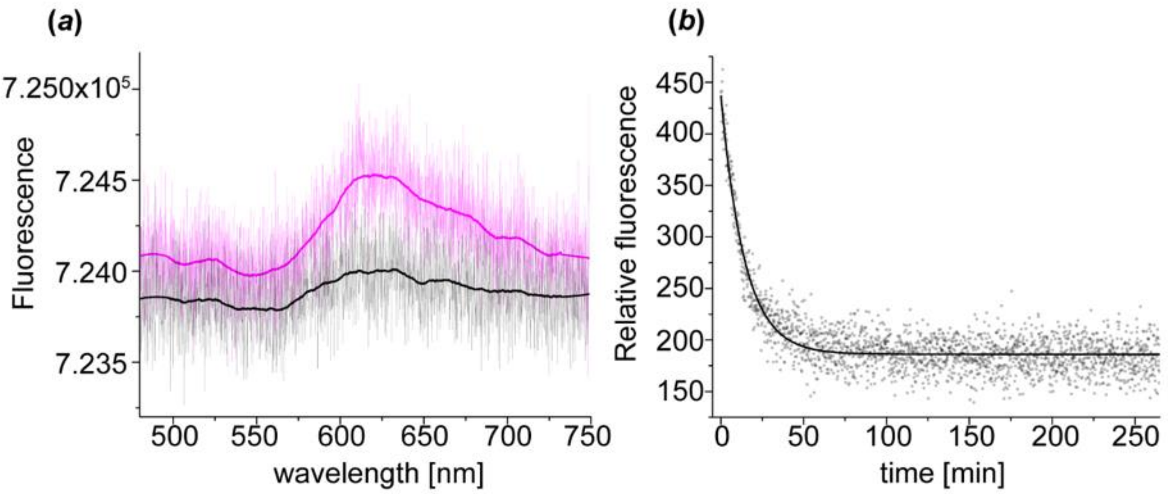

Figure S1 Fluorescence measurements. (*a*) Exemplary raw and smoothened accumulated optical spectra taken at the beginning (t_0, magenta) and the end of the kinetic series (t = 265 min, black) for the "5" type of setup indicated in Fig. 2(*d*). (*b*) Exemplary plot showing decay of the fluorescence signal for the "3" type of setup indicated in Fig. 2(*d*) plotted as the absolute difference between the number of counts at 615 nm with 550 nm (dots) fitted to a single exponential decay model (solid line), from which a $t_{0.5}$ = 13.7 minutes was obtained.

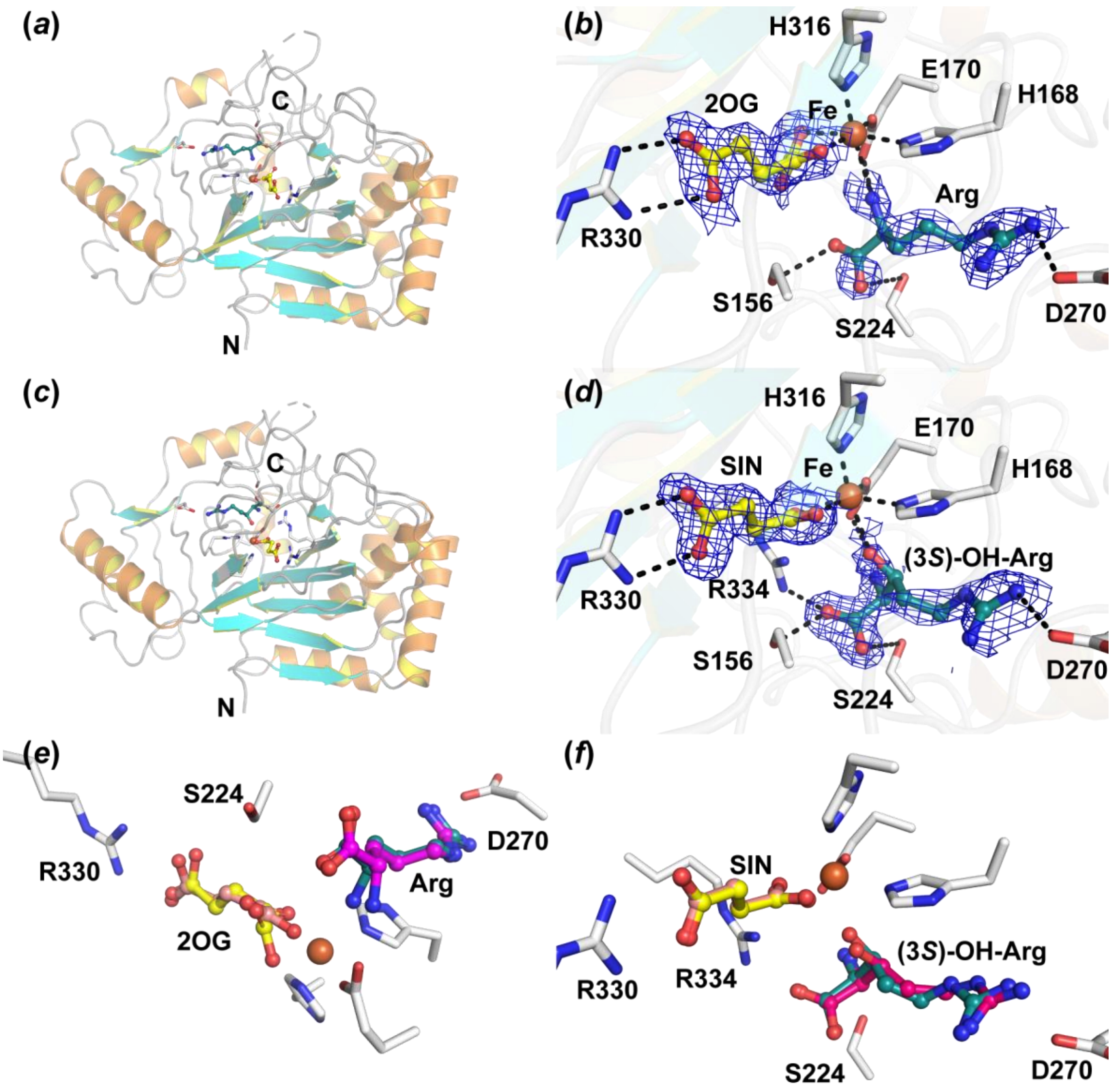

Figure S2 Structural comparison of the anaerobic and O_2 exposed VioC structures. (*a*) Ribbons representation and (*b*) close-up view of the active site from the VioC:Fe:2OG:Arg complex structure reported in this study (PDB: 6Y0N). The N- and C-termini are labelled as N and C, respectively. Alpha-helices and beta-strands are shown in gold and cyan, respectively. The arginine substrate, 2OG cosubstrate and coordinating residues are shown in sticks. Panel (*b*) shows the composite omit map to 1.86 Å resolution displayed at 0.7 σ contour level, carved around the arginine substrate and 2OG. (*c*) Ribbons representation and (*d*) close-up view of the active site of VioC:Fe:succinate:(3*S*)-hydroxy-l-Arg complex after dioxygen exposure (PDB: 6Y12). Panel (*d*) shows the composite omit map to 1.70 Å resolution displayed at 0.7 σ contour level, carved around the (3*S*)-hydoxy-l-Arg and succinate. (*e*) Superimposition of the cryogenic (magenta, and salmon, PDB: 6ALM) and serial room temperature (cyan, and yellow, PDB: 6Y0N, this study) VioC:Fe:2OG:Arg complex structures reveals small apparent differences in the bidentate coordination of the metal by 2OG, as well as a change in the conformation of the arginine substrate (including the α-amino group), possibly reflecting the flexible nature of the VioC active site (Mitchell *et al.*, 2017). (*f*) Superimposition of the cryogenic (magenta

and salmon, PDB: 2WBP) and serial room temperature (cyan and yellow) VioC:Fe:succinate:(3*S*)-hydroxy-l-Arg complex (PDB: 6Y12, this study) structures reveals small apparent differences in the conformation of (3*S*)-hydroxy-l-Arg, whereas the binding of succinate is consistent with that in the reported cryogenic structure (Helmetag *et al*., 2009; Mitchell *et al*., 2017).
